# Appendiceal-origin pseudomyxoma peritonei masked by culture-positive neutrocytic ascites: a case report

**DOI:** 10.1093/omcr/omag084

**Published:** 2026-06-08

**Authors:** Imane Chenfouh, Kamel Nabi, Amina Diaf, Riadh Gaffaf, Aya Mahdi

**Affiliations:** Chaumont Hospital Centre, Department of Medicine 3, 2 rue Jeanne d'Arc, 52000 Chaumont, Haute-Marne, France; Chaumont Hospital Centre, Department of Medicine 3, 2 rue Jeanne d'Arc, 52000 Chaumont, Haute-Marne, France; Chaumont Hospital Centre, Department of Medicine 3, 2 rue Jeanne d'Arc, 52000 Chaumont, Haute-Marne, France; Chaumont Hospital Centre, Department of Medicine 3, 2 rue Jeanne d'Arc, 52000 Chaumont, Haute-Marne, France; Chaumont Hospital Centre, Department of Medicine 3, 2 rue Jeanne d'Arc, 52000 Chaumont, Haute-Marne, France

**Keywords:** pseudomyxoma peritonei, appendiceal mucinous neoplasm, computed tomography, mucinous ascites, bacterial peritonitis

## Abstract

Pseudomyxoma peritonei (PMP) is a rare peritoneal surface malignancy, most often of appendiceal origin. Diagnosis may be delayed when symptoms are indolent or when ascitic fluid studies suggest infection. A 65-year-old man presented with 1 year of progressive abdominal distension and 15 days of epigastric pain. He was haemodynamically stable and afebrile. Paracentesis showed cloudy exudative ascites with marked neutrophilia (17 900 cells/mm^3^; 52% neutrophils). Culture grew *Escherichia coli* and cytology was negative. Portal venous phase CT demonstrated complex hyperattenuating ascites, diffuse peritoneal implants with bulky omental caking, visceral scalloping and an abnormal enhancing appendix. Ultrasound-guided omental core biopsies confirmed high-grade mucinous carcinoma peritonei without signet-ring cells, supporting an appendiceal-type primary. The patient declined surgical exploration and died shortly after readmission with bowel perforation and abscess. This case underscores that characteristic CT findings should prompt tissue confirmation and explicit tumour grading even when ascites appears infectious.

## Introduction

Mucinous appendiceal neoplasms are rare mucin-rich epithelial tumours with a spectrum of behaviour ranging from low-grade lesions to invasive adenocarcinoma [1, 2]. Perforation can disseminate mucin and tumour epithelium throughout the peritoneal cavity, producing pseudomyxoma peritonei (PMP) [2, 3]. Because prognosis and treatment differ markedly between low-grade and high-grade disease, explicit histopathological classification of the peritoneal component, including documentation of signet-ring cells, is essential [1, 2, 4]. Neutrocytic ascites in malignancy may mimic bacterial peritonitis and delay diagnosis [5]. We report a biopsy-confirmed high-grade mucinous carcinoma peritonei in which culture-positive neutrocytic ascites initially supported bacterial peritonitis, delaying recognition of underlying PMP.

## Case report

A 65-year-old man with hypertension, diabetes mellitus, prior non–ST elevation myocardial infarction treated with coronary stenting, obstructive sleep apnoea on continuous positive airway pressure, obesity (BMI 31 kg/m^2^) and an umbilical hernia presented with 15 days of persistent epigastric pain and a 1-year history of progressive abdominal distension. He was not up to date with colorectal cancer screening and had no family history of gastrointestinal malignancy.

On admission he was stable (blood pressure 110/70 mmHg, heart rate 90 beats/min, temperature 37°C, respiratory rate 12 breaths/min, oxygen saturation 96% on room air) and had no clinical features of septic shock. Abdominal examination showed distension with epigastric and peri-umbilical tenderness without guarding or rigidity. Laboratory tests demonstrated leukocytosis (10 970/mm^3^), C-reactive protein 221 mg/l, microcytic anaemia (haemoglobin 10 g/dl; MCV 73.7 fl), thrombocytosis (632 × 10^9^/l), hypoalbuminaemia (24 g/l) and prothrombin time 65%. Tumour markers were elevated (CEA 30.43 ng/ml; CA19–9238 U/ml).

Bedside diagnostic paracentesis was performed using sterile technique. Image guidance was not required because of abundant free fluid. Ascites was cloudy and exudative (protein 53.2 g/l) with 17 900 nucleated cells/mm^3^ (52% neutrophils; estimated polymorphonuclear count approximately 9300/μl). Gram stain was negative but culture grew *Escherichia coli*. Blood cultures were negative and antibiotics started.

Cytology of ~ 10 ml yellow haemorrhagic fluid processed by cytocentrifugation showed no malignant cells.

Portal venous phase contrast-enhanced CT demonstrated large-volume complex/hyperattenuating ascites, diffuse peritoneal implants with bulky omental caking and scalloping of the liver and spleen. Disease extended into an ~ 8 cm umbilical hernia, and the appendix showed abnormal enhancement, favouring PMP with an appendiceal-pattern primary (Fig. 1).

**Figure 1:**
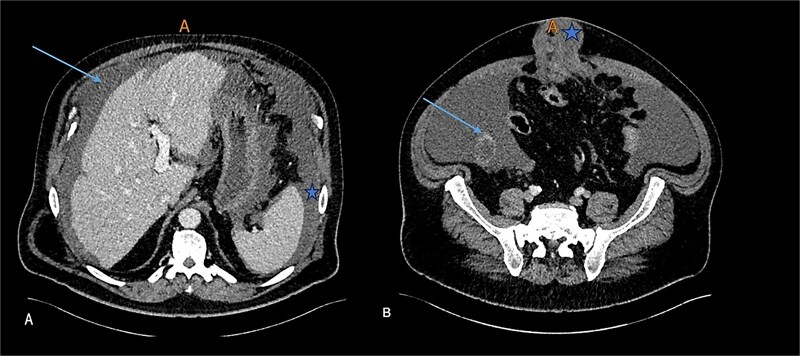
Portal venous phase contrast-enhanced CT at presentation. (A) Upper abdomen shows scalloping of the anterior hepatic margin (arrow) and splenic surface (asterisk) due to mucinous/complex ascites. (B) Lower abdomen/pelvis shows an abnormally enhancing appendix (arrow) with hyperattenuating complex ascites and extension into the umbilical hernia sac (asterisk), supporting appendiceal-origin PMP.

Ultrasound-guided transcutaneous 18-gauge core biopsies from the omental cake and an umbilical metastasis were obtained, no immediate iatrogenic complications were observed. Histology showed abundant extracellular mucin in large pools within desmoplastic/sclerotic stroma containing infiltrative gland-forming and cord-like epithelium with marked atypia and rare mitoses, consistent with high-grade mucinous carcinoma peritonei without signet-ring cells (Fig. 2). Immunohistochemistry supported a lower gastrointestinal/appendiceal-type origin (CK7+, CDX2+, CK20 heterogeneous, SATB2+, MUC2+). Mismatch repair proteins were intact. Somatic testing identified TP53, GNAS and KRAS mutations (Fig. 3).

**Figure 2:**
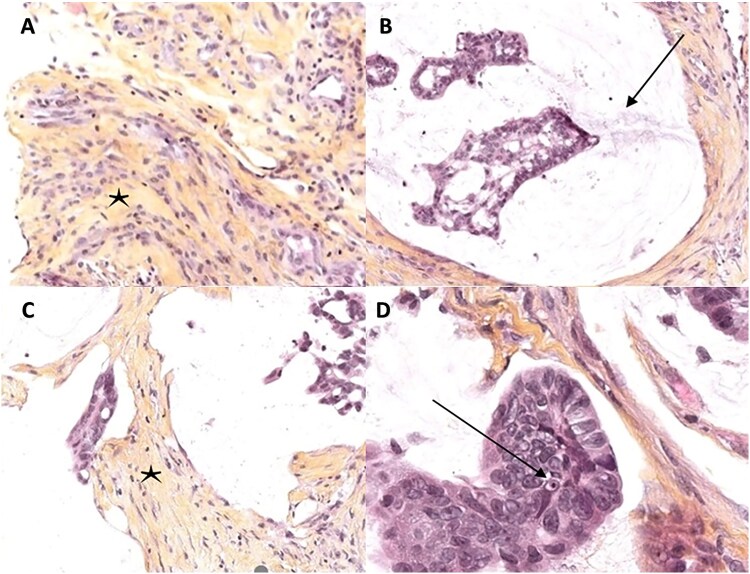
Haematoxylin and eosin-stained sections from ultrasound-guided core biopsies. (A) × 10: Desmoplastic/sclerotic stroma (asterisk) with infiltrative epithelial elements. (B) × 10: Large extracellular mucin pool (arrow) containing clusters of neoplastic epithelium. (C) × 10: Additional fibrous stroma (asterisk) adjacent to mucin and tumour epithelium. (D) × 40: Infiltrative glandular/cord-like tumour structures with marked atypia and rare mitoses (arrow).

**Figure 3:**
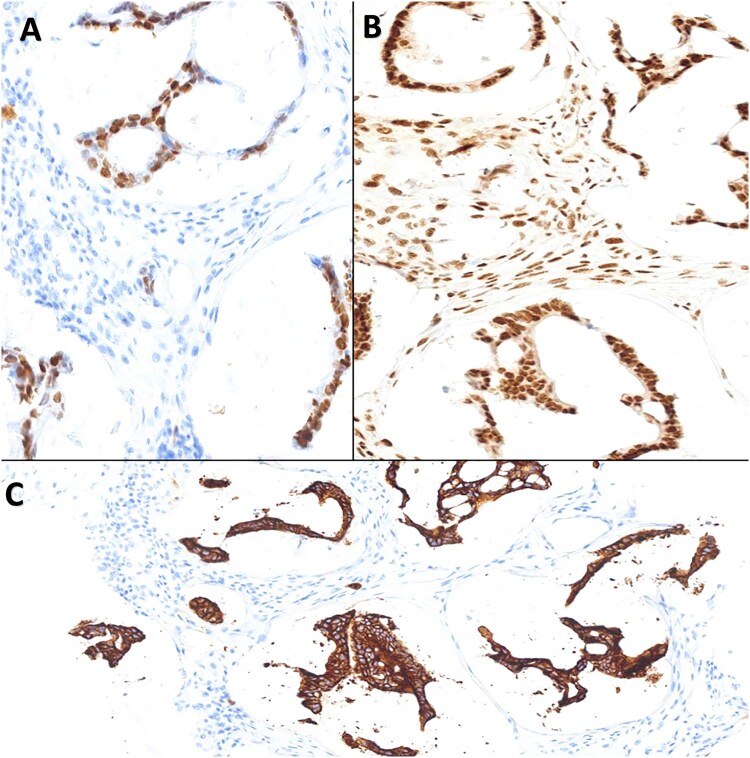
Representative immunohistochemistry from omental core biopsy (original magnification ×40). (A) CDX2 shows diffuse nuclear staining of tumour cells within extracellular mucin pools. (B) MLH1 shows retained nuclear expression in tumour cells, with positive internal control in adjacent stromal cells, consistent with intact mismatch repair protein expression. (C) CK7 shows diffuse cytoplasmic staining of tumour cells lining glandular and cord-like structures within mucin pools.

CT suggested extensive multi-quadrant peritoneal tumour burden. A formal intraoperative Peritoneal Cancer Index (PCI) was not obtained because the patient declined exploratory surgery, therefore, extent was assessed radiologically. Following multidisciplinary discussion, staging with FDG PET/CT and systemic therapy planning (FOLFOX-based) were arranged.

After discharge, he re-presented 12 days later with dyspnoea and clinical deterioration due to pulmonary embolism and acute decompensated heart failure. Repeat CT demonstrated small-bowel perforation with a large walled-off intra-abdominal abscess (Fig. 4). Because there were no immediate post-procedural signs of visceral injury and the perforation occurred after a 12-day interval, an acute biopsy-related perforation was considered less likely, however, a delayed procedure-related complication could not be definitively excluded. Alternative explanations included tumour-related bowel compromise and/or progression of intra-abdominal infection in advanced peritoneal disease.

**Figure 4:**
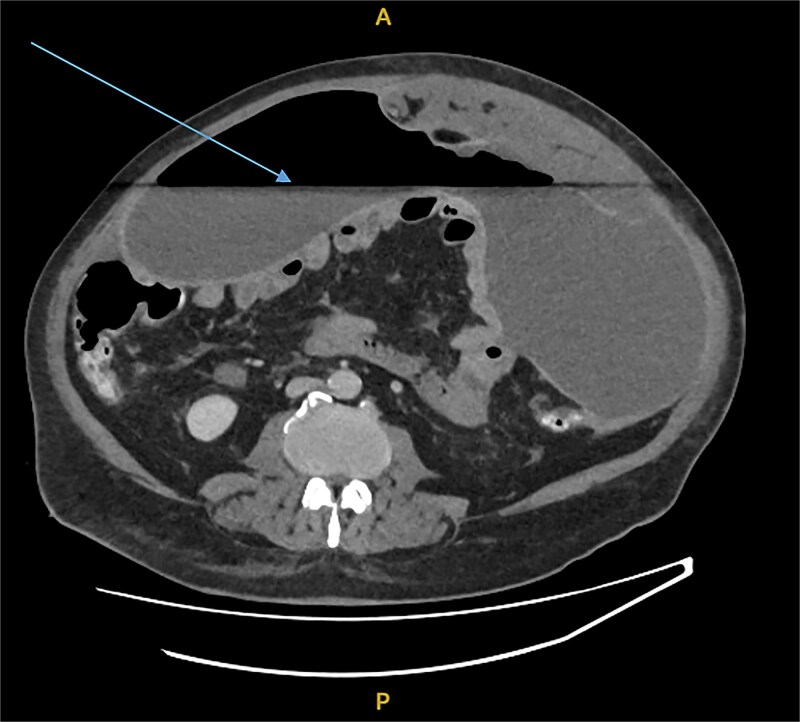
Portal venous phase contrast-enhanced CT at readmission showing a large intraperitoneal air–fluid collection (arrow) with internal gas and dependent fluid level, consistent with a walled-off abscess in the setting of bowel perforation.

The patient declined surgery and chose do-not-resuscitate status. He was managed conservatively with image-guided drainage, antibiotics, supportive care and symptom control. He developed acute decompensated heart failure with pulmonary embolism and died a few days later under palliative care. His decline reflected multiple contributing conditions rather than one isolated cause.

## Discussion

The diagnosis of PMP relies primarily on cross-sectional imaging, with contrast-enhanced CT remaining the modality of choice. CT demonstrates mucinous ascites, peritoneal implants and often an appendiceal-pattern primary, while defining disease distribution for management. Visceral scalloping is a key sign that supports mucin-rich ascites rather than simple fluid [3]. Malignant ascites may meet neutrocytic criteria despite the absence of true infection, so characteristic CT findings should still prompt further investigation even when fluid studies suggest infection [5].

Histopathologic confirmation is essential. PSOGI (Peritoneal Surface Oncology Group International) classifies mucinous carcinoma peritonei as low-grade or high-grade, with or without signet-ring cells, because prognosis and treatment differ substantially [1, 2]. Tumour markers contribute to risk stratification: elevated CA19–9 and CEA correlate with high-grade disease and recurrence, while normal preoperative CEA predicts successful cytoreduction (CC0–1) [4]. Core biopsy in our patient showed high-grade mucinous carcinoma peritonei without signet-ring cells, acknowledging sampling limitations. Immunohistochemistry supported an appendiceal-type lower gastrointestinal origin, with CK20, CDX2, SATB2 and MUC2 expression supporting this interpretation [6]. Molecular profiling may provide additional biological context, as KRAS/GNAS mutations are common in low-grade disease and TP53 alterations are more frequent in high-grade tumours [7]. Although the immunoprofile supported a lower gastrointestinal/appendiceal-type primary, definitive confirmation ideally requires histology of the appendix. Here, appendiceal origin was inferred from imaging and immunophenotype.

Resectability is guided by tumour burden, commonly assessed intraoperatively using the PCI, and survival is strongly linked to complete cytoreduction (CC0/CC1) [8]. Current guidelines recommend cytoreductive surgery with hyperthermic intraperitoneal chemotherapy (CRS/HIPEC) as the standard approach for resectable PMP due to survival benefit. In our patient, extensive bulky disease on CT, major comorbidities and rapid complications precluded CRS/HIPEC, and exploratory surgery was declined, so PCI was not measured. For unresectable disease, systemic chemotherapy such as FOLFOX may be used, and bevacizumab has been suggested in colorectal-type peritoneal disease, although robust prospective evidence remains limited [9, 10]. When CT suggests PMP (mucinous ascites rather than simple fluid, peritoneal implants, visceral scalloping), diagnostic work-up should not be delayed by negative cytology or neutrophilic ascites suggestive of infection. Clinicians should pursue prompt tissue confirmation with grading and early referral to a peritoneal surface malignancy team, while recognising that comorbidity and tumour burden may preclude CRS/HIPEC.
